# Data on spatio-temporal patterns of wild fruit harvest from the economically important palm *Mauritia flexuosa* in the Peruvian Amazon

**DOI:** 10.1016/j.dib.2018.07.045

**Published:** 2018-07-27

**Authors:** Bryan A. Endress, Michael P. Gilmore, Victor H. Vargas Paredes, Christa M. Horn

**Affiliations:** a Eastern Oregon Agriculture and Natural Resource Program, Oregon State University, La Grande, OR 97850, United States; b School of Integrative Studies, George Mason University, Fairfax, VA 22030, United States; c Institute for Conservation Research, San Diego Zoo Global, Escondido, CA 92027, United States

## Abstract

These data are the foundation of the analyses and results published in the article “Spatio-temporal patterns of *Mauritia flexuosa* fruit extraction in the Peruvian Amazon: Implications for conservation and sustainability” (Horn et al., 2018) [1]. Here we include data on the volume of *M. flexuosa* fruit arriving in the city of Iquitos, Peru from the surrounding region. This includes the amount of fruit (in sacks and kg), the date of entry into Iquitos, the point of embarkation (watershed and coordinates), the method of transportation and the point of entry into Iquitos. Data is provided in a number of formats, including data tables, Google Earth KML files and summary tables by watershed and/or month.

**Specifications Table**

**Table:** 

Subject area	Geography, natural resources, social sciences, conservation, forestry
More specific subject area	Applied ecology and conservation
Type of data	Table (csv), Text file, KMZ (Google Earth)
How data was acquired	Surveys, GPS, GIS
Data format	Raw and aggregated by month for locations and watersheds
Experimental factors	None
Experimental features	None
Data source location	Loreto, Peru
Data accessibility	Data is with this article

**Value of the data**

The data presented provides baseline numbers of *M. flexuosa* (aguaje) fruit entering the market of Iquitos, Loreto, Peru, over one year (2012–2013) which can be compared to future studies to document change in extraction levels.

• Further analyses of the data can be used by policy, conservation and resource management entities to prioritize geographic areas or communities for outreach efforts focused on sustainable harvest of aguaje and guide the timing of those efforts.
• Data can be incorporated into other geographical studies focused on the regional transportation system or other natural resources for further analysis.
• Data for individual communities can provide insights into market interaction and resource extraction decisions of those communities.

## 1 Data

In the Amazon Basin, some non-timber forest products (NTFP), such as the ecologically and economically important palm *Mauritia flexuosa*, are extracted intensively and across large areas. The ecological effect of harvest is unclear [1]. Fruit is harvested from wild populations of *M. flexuosa* and is eaten directly or processed into juice, ice cream, and other food products. Because adult palms can grow above 30 m in height, harvest is primarily destructive and fruiting females are cut down in order to harvest the fruit. The demand for fruit is driven by the city of Iquitos, the commercial center of Loreto, and the largest consumer of *M. flexuosa* fruit in the Amazon [2]. Despite decades of concern about overharvesting, and the ecological implications of harvest, the scale and scope of *M. flexuosa* extraction remains unclear [3]. To better understand the magnitude of *M. flexuosa* harvest in the region, we quantified the amount of *M. flexuosa* fruit entering the Iquitos market, traced its source and documented spatial and temporal patterns of extraction across the region. Specifically, this data article includes the following:

1. Downloadable CSV file (Appendix A) that includes all records of *M. flexuosa* entering the Iquitos market during the study period, including date, number of sacks, weight, origin (watershed, UTM coordinates, name of embarkation village or location), mode of transportation, how the data was collected, and any notes associated with the record.
2. Tables that summarize *M. flexuosa* extraction by watershed and month.
3. Google Earth file (KML), that visually displays the data (Appendix B).

## 2 Experimental design, materials and methods

Data were collected daily between April 2012 and March 2013 at the major points of entry into Iquitos: the private ports of Don Jose and Sofy; the public ports of Productores, Belen, Pescaderos, and Masusa within Iquitos; the public ports of Bellavista Nanay, Morona Cocha, Pampa Chica, and Nina Rumi on the city outskirts; and the bus stop in Belen. To determine the amount of *M. flexuosa* and where it came from, we interviewed boat crews. We also reviewed log books of boats (*colectivos* and *lanchas*) that maintain detailed records of passengers, their point of embarkation, and the amounts of the products that they are transporting, including *M. flexuosa*. People travelling via *peque-peques* (small, slow motorized boats) and canoes do not keep records, and we directly interviewed operators at ports about the *M. flexuosa* fruit they brought to market. For *M. flexuosa* entering Iquitos by bus, we collaborated with the bus drivers who reported incoming fruit and its source to a supervisor who recorded the information on datasheets we provided. We used an average fruit sack weight of 36 kg (based on data collected at ports) to convert the number of sacks counted to weight [1]. Data were incorporated into GIS to visually display sources of incoming *M. flexuosa* fruit. This allowed us to quantify the spatio-temporal patterns of harvest, from which watersheds fruit was coming from, and which communities and locations were providing the most fruit to the market and when (Table 1, Table 2, Table 3, Table 4, Table 5, Table 6, Table 7, Table 8). Additional details and specifics on methods are described by Horn et al. [1].

**Table 1:** Top 10 sources of *M. flexuosa* in the Amazonas watershed. We recorded a total of 109 source locations of fruit in the Amazonas watershed over the 12-month study.

Rank	Embarkation point	Number of shipments	Maximum shipment		Mean fruit per shipment		Total fruit imported		Watershed imports (%)	Total annual imports (%)
			Sacks	mT	Sacks	mT	Sacks	mT		
1	Itaya	635	–	5.4	22.3	0.8	14,146	509.2	40.2	6.2
2	Amazonas	118	–	4.5	56.4	2.0	6652	239.5	18.9	2.9
3	Recreo	54	135	4.9	25.9	0.9	1396	50.3	4.0	0.6
4	Maniti	49	80	2.9	22.3	0.8	1093	39.3	3.1	0.5
5	Quebrada Yanayacu	91	30	1.1	11.3	0.4	1031	37.1	2.9	0.5
6	Limón	189	16	0.6	5.0	0.2	942	33.9	2.7	0.4
7	Santa Cecilia	26	120	4.3	34.4	1.2	894	32.2	2.5	0.4
8	Puerto Alegría	119	20	0.7	7.2	0.3	860	31.0	2.4	0.4
9	San Juan de Munich	110	35	1.3	6.0	0.2	662	23.8	1.9	0.3
10	Aucayo	68	28	1.0	9.5	0.3	645	23.2	1.8	0.3
Top 10 Cumulative		1459	–	–	19.4	0.7	28,320	1019.5	80.4	12.4
Amazonas Totals		2340	150.86	5.4	15.0	0.5	35,207	1267.5	100	15.4

**Table 2:** Top 10 sources of *M. flexuosa* in the Bajo Amazonas watershed. A total of 38 source locations of fruit were recorded in this watershed over the 12-month study.

Rank	Embarkation point	Number of shipments	Maximum shipment		Mean fruit per shipment		Total fruit imported		Watershed imports (%)	Total annual imports (%)
			Sacks	mT	Sacks	mT	Sacks	mT		
1	Apayacu	142	123	4.4	35.1	1.3	4980	179.3	29.3	2.2
2	Yanashi	89	130	4.7	31.0	1.1	2756	99.2	16.2	1.2
3	Bajo Amazonas	71	50	1.8	34.2	1.2	2427	87.4	14.3	1.1
4	San Gregorio	76	104	3.7	31.8	1.1	2414	86.9	14.2	1.1
5	Islandia	29	131	4.7	34.8	1.3	1009	36.3	5.9	0.4
6	Oran	66	68	2.4	13.2	0.5	872	31.4	5.1	0.4
7	Canton	37	66	2.4	18.4	0.7	680	24.5	4.0	0.3
8	Orosa	7	50	1.8	30.3	1.1	212	7.6	1.2	0.1
9	San Pedro	6	120	4.3	28.7	1.0	172	6.2	1.0	0.1
10	Colonia	14	27	1.0	11.4	0.4	160	5.8	0.9	0.1
Top 10 cumulative		537	–	–	29.2	1.1	15,681	564.5	92.1	6.9
Watershed Total		663	131	4.7	25.7	0.9	17,023	612.8	100	7.5

**Table 3:** Source locations of *M. flexuosa* fruit in the Bajo Marañón watershed. Nine source locations of fruit were recorded over the 12-month study.

Rank	Embarkation point	Number of shipments	Maximum shipment		Mean fruit per shipment		Total fruit imported		Watershed imports (%)	Total annual imports (%)
			Sacks	mT	Sacks	mT	Sacks	mT		
1	Nauta	245	210	7.6	53.6	1.9	13,123	472.4	81.0	5.8
2	Yanayacu Pucate	35	200	7.2	79.9	2.9	2798	100.7	17.3	1.2
3	Monte Carmelo	12	15	0.5	7.3	0.3	88	3.2	0.5	0.0
4	Nuevo Miraflores	1	72	2.6	72.0	2.6	72	2.6	0.4	0.0
5	Solteritos	6	15	0.5	9.7	0.3	58	2.1	0.4	0.0
6	Bagazán	2	30	1.1	27.0	1.0	54	1.9	0.3	0.0
7	Quebrada Cumapa	1	5	0.2	5.0	0.2	5	0.2	0.0	0.0
8	Puerto Perú	3	5	0.2	3.3	0.1	10	0.4	0.1	0.0
9	20 de Enero	1	3	0.1	3.0	0.1	3	0.1	0.0	0.0
Top 9 cumulative		306	–	–	53.0	1.9	16,211	583.6	100.0	7.1
Watershed Total		306	210	7.6	53.0	1.9	16,211	583.6	100	7.1

**Table 4:** Top 10 source locations of *M. flexuosa* fruit in the Bajo Ucayali watershed. A total of 18 source locations of fruit were recorded over the 12-month study.

Rank	Embarkation point	Number of shipments	Maximum shipment		Mean fruit per shipment		Total fruit imported		Watershed imports (%)	Total annual imports (%)
			Sacks	mT	Sacks	mT	Sacks	mT		
1	Requena	72	180	6.5	50.1	1.8	3606	129.8	66.0	1.6
2	Jenaro Herrera	48	116	4.2	20.1	0.7	966	34.8	17.7	0.4
3	Libertad	11	50	1.8	16.5	0.6	181	6.5	3.3	0.1
4	Puerto Miguel	7	62	2.2	22.4	0.8	157	5.7	2.9	0.1
5	Capitan Clavero	12	20	0.7	9.5	0.3	114	4.1	2.1	0.1
6	Sapuena	3	53	1.9	32.7	1.2	98	3.5	1.8	0.0
7	Bretaña	2	72	2.6	43.5	1.6	87	3.1	1.6	0.0
8	Castaña	4	22	0.8	13.5	0.5	54	1.9	1.0	0.0
9	Ucayali	1	42	1.5	42.0	1.5	42	1.5	0.8	0.0
10	Santa Elena	1	40	1.4	40.0	1.4	40	1.4	0.7	0.0
Top 10 cumulative		161	–	–	33.2	1.2	5345	192.4	97.8	2.3
Watershed Total		169	180	6.5	32.3	1.2	5467	196.8	100	2.4

**Table 5:** Top 10 source locations of *M. flexuosa* fruit in the Medio Bajo Marañón watershed. A total of 45 source locations of fruit were recorded over the 12-month study.

Rank	Embarkation point	Number of shipments	Maximum shipment		Mean fruit per shipment		Total fruit imported		Watershed imports (%)	Total annual imports (%)
			Sacks	mT	Sacks	mT	Sacks	mT		
1	San Roque	127	672	24.2	171.0	6.2	21,717	781.8	19.0	9.5
2	Santa Rita de Castilla	192	360	13.0	77.9	2.8	14,963	538.7	13.1	6.6
3	Santa Rosa de Lagarto	61	524	18.9	165.2	5.9	10,076	362.7	8.8	4.4
4	Cuninico	106	326	11.7	89.8	3.2	9520	342.7	8.3	4.2
5	San José de Parinari	75	431	15.5	102.4	3.7	7683	276.6	6.7	3.4
6	Roca Fuerte	105	430	15.5	71.6	2.6	7519	270.7	6.6	3.3
7	Parinari	99	333	12.0	67.5	2.4	6684	240.6	5.8	2.9
8	Alianza	62	375	13.5	86.9	3.1	5390	194.0	4.7	2.4
9	Buena Vista Jerusalen	57	287	10.3	76.6	2.8	4369	157.3	3.8	1.9
10	San José de Saramuro	79	163	5.9	38.2	1.4	3014	108.5	2.6	1.3
Top 10 cumulative		963	–	–	94.4	3.4	90,935	3273.7	79.5	39.9
Watershed Total		1519	672	24.2	75.3	2.7	114,361	4117.0	100	50.2

**Table 6:** Top 10 source locations of *M. flexuosa* fruit in the Napo watershed. 37 source locations of fruit were recorded over the 12-month study.

Rank	Embarkation point	Number of shipments	Maximum shipment		Mean fruit per shipment		Total fruit imported		Watershed imports (%)	Total annual imports (%)
			Sacks	mT	Sacks	mT	Sacks	mT		
1	Mazan	392	213	7.7	31.0	1.1	12,145	437.2	70.2	5.3
2	Tigrillo	27	50	1.8	20.2	0.7	546	19.7	3.2	0.2
3	Bagazán	20	119	4.3	25.9	0.9	517	18.6	3.0	0.2
4	Nuñez Cocha	18	60	2.2	21.7	0.8	391	14.1	2.3	0.2
5	Santa Lucía	12	74	2.7	32.0	1.2	384	13.8	2.2	0.2
6	Napo	61	100	3.6	6.0	0.2	369	13.3	2.1	0.2
7	San Pedro de Mangua	16	60	2.2	21.0	0.8	336	12.1	1.9	0.1
8	Mangua	20	44	1.6	14.1	0.5	283	10.2	1.6	0.1
9	Nuevo Progreso	11	90	3.2	23.8	0.9	262	9.4	1.5	0.1
10	Yurac Yacu	17	54	1.9	14.1	0.5	240	8.6	1.4	0.1
Top 10 cumulative		594	–	–	26.0	0.9	15,471	556.9	89.4	6.8
Watershed Total		736	213	7.7	23.5	0.8	17,309	623.1	100	7.6

**Table 7:** Top 10 source locations of *M. flexuosa* fruit in the Tigre watershed. 16 source locations of fruit were recorded over the 12-month study.

Rank	Embarkation point	Number of shipments	Maximum shipment		Mean fruit per shipment		Total fruit imported		Watershed imports (%)	Total annual imports (%)
			Sacks	mT	Sacks	mT	Sacks	mT		
1	Nueva York	109	600	21.6	146.7	5.3	15,985	575.5	71.6	7.0
2	Monte Verde	27	143	5.1	70.4	2.5	1900	68.4	8.5	0.8
3	Miraflores	26	200	7.2	61.2	2.2	1590	57.2	7.1	0.7
4	Bellavista	27	158	5.7	52.8	1.9	1425	51.3	6.4	0.6
5	Piura	7	130	4.7	59.1	2.1	414	14.9	1.9	0.2
6	Santa Cruz	8	117	4.2	47.4	1.7	379	13.6	1.7	0.2
7	San Jorge	9	82	3.0	40.9	1.5	368	13.2	1.6	0.2
8	Nuevo Paraiso	1	64	2.3	64.0	2.3	64	2.3	0.3	0.0
9	Paraiso	2	40	1.4	28.5	1.0	57	2.1	0.3	0.0
10	Quebrada Nahuapa	2	45	1.6	24.0	0.9	48	1.7	0.2	0.0
Top 10 cumulative		218	–	–	102.0	3.7	22,230	800.3	99.5	9.8
Watershed Total		224	600	21.6	99.7	3.6	22,334	804.0	100	9.8

**Table 8:** Monthly pattern of *M. flexuosa* fruit shipments to Iquitos by watershed. Values are in metric tons (mT).

	2012									2013				
Watershed	April	May	June	July	Aug.	Sept.	Oct.	Nov.	Dec.	Jan.	Feb.	Mar.	Total	Embarkation points (#)
Huallaga	0.0	0.0	0.0	0.0	0.0	0.0	0.0	0.0	0.0	0.0	0.0	1.1	1.1	1
Bajo Ucayali	11.6	13.1	14.7	9.3	13.9	7.2	11.6	41.3	33.2	10.6	12.6	17.9	196.8	18
Tigre	3.6	0.4	3.0	2.9	14.9	59.7	38.6	134.1	172.7	152.5	131.9	89.6	804.0	16
Medio Bajo Marañón	105.6	9.9	0.0	0.0	22.5	186.8	542.7	680.2	680.7	668.3	606.6	610.2	4113.5	45
Bajo Marañón	23.6	22.4	1.9	2.2	18.8	90.0	73.8	61.9	91.8	79.2	46.8	74.7	587.1	9
Amazonas	33.2	110.8	279.4	386.8	364.7	91.9	0.0	0.0	0.0	0.0	0.0	0.6	1267.5	109
Bajo Amazonas	47.0	156.5	111.4	138.9	119.5	32.5	0.0	0.0	0.0	0.0	0.5	6.4	612.8	38
Napo	6.7	26.1	77.1	105.9	203.4	168.2	20.3	0.0	0.0	1.1	7.8	6.6	623.1	37
Total Imported	231.2	339.2	487.5	646.0	757.7	636.4	686.9	917.5	978.4	911.7	806.2	807.2	8205.9	
Embarkation Points (#)	103	109	108	109	114	120	37	46	40	43	40	50		273

## Appendix B Supplementary materials

The following KMZ files contain the Google maps of the most important areas described in this article.

Map KMZ file containing the Google map.

## Footnotes

Appendix A Supplementary data associated with this article can be found in the online version at https://doi.org/10.1016/j.dib.2018.07.045.

Appendix B Supplementary data associated with this article can be found in the online version at https://doi.org/10.1016/j.dib.2018.07.045. These data include Google maps of the most important areas described in this article.
