# Supplementary material for: Data on spatio-temporal patterns of wild fruit harvest from the economically important palm Mauritia flexuosa in the Peruvian Amazon
Source: Data Brief. 2018 Jul 27;20:132–9. doi: 10.1016/j.dib.2018.07.045 (PMC6091237; doi:10.1016/j.dib.2018.07.045)
Supplement: Supplementary file 1 — Transparency document [file mmc1.docx]

Conflict of Interest and Authorship Conformation Form

Please check the following as appropriate:

**X**

- All authors have participated in (a) conception and design, or analysis and interpretation of the data; (b) drafting the article or revising it critically for important intellectual content; and (c) approval of the final version.
- This manuscript has not been submitted to, nor is under review at, another journal or other publishing venue.
- The authors have no affiliation with any organization with a direct or indirect financial interest in the subject matter discussed in the manuscript
- The following authors have affiliations with organizations with direct or indirect financial interest in the subject matter discussed in the manuscript:

Author’s name Affiliation
